# Hypervirulent *Klebsiella pneumoniae* in Cryptogenic Liver Abscesses, Paris, France

**DOI:** 10.3201/eid2402.170957

**Published:** 2018-02

**Authors:** Benjamin Rossi, Maria Ludovica Gasperini, Véronique Leflon-Guibout, Alice Gioanni, Victoire de Lastours, Geoffrey Rossi, Safi Dokmak, Maxime Ronot, Olivier Roux, Marie-Hélène Nicolas-Chanoine, Bruno Fantin, Agnès Lefort

**Affiliations:** Hôpital Beaujon, Clichy, France (B. Rossi, M.L. Gasperini, V. Leflon-Guibout, A. Gioanni, V. de Lastours, G. Rossi, S. Dokmak, M. Ronot, O. Roux, M.-H. Nicolas-Chanoine, B. Fantin, A. Lefort);; Université Paris Diderot, Paris, France (V. de Lastours, M. Ronot, Marie-Hélène Nicolas-Chanoine, B. Fantin, A. Lefort)

**Keywords:** *Klebsiella pneumoniae*, bacteria, hypervirulent, cryptogenic liver abscesses, Paris, France

## Abstract

Liver abscesses containing hypervirulent *Klebsiella pneumoniae* have emerged during the past 2 decades, originally in Southeast Asia and then worldwide. We hypothesized that hypervirulent *K. pneumoniae* might also be emerging in France. In a retrospective, monocentric, cohort study, we analyzed characteristics and outcomes for 199 consecutive patients in Paris, France, with liver abscesses during 2010−2015. We focused on 31 patients with abscesses containing *K. pneumoniae*. This bacterium was present in most (14/27, 52%) cryptogenic liver abscesses. Cryptogenic *K. pneumoniae* abscesses were more frequently community-acquired (p<0.00001) and monomicrobial (p = 0.008), less likely to involve cancer patients (p<0.01), and relapsed less often (p<0.01) than did noncryptogenic *K. pneumoniae* liver abscesses. *K. pneumoniae* isolates from cryptogenic abscesses belonged to either the K1 or K2 serotypes and had more virulence factors than noncryptogenic *K. pneumoniae* isolates. Hypervirulent *K. pneumoniae* are emerging as the main pathogen isolated from cryptogenic liver abscesses in the study area.

## Abstract 2

Europe might have an epidemic of infections with these bacteria similar to that in Asia in the early 2000s.

Pyogenic liver abscesses are common intra-abdominal infections (*1*–*4*), associated with a substantial severity (*5*). Most (30%–70%) pyogenic liver abscesses result from a biliary origin (*6*), followed by a portal origin (10%−20%) complicating intra-abdominal diseases, such as appendicitis, diverticulitis, infected gastrointestinal tumors, or chronic inflammatory bowel disease. Less frequently, pyogenic liver abscesses might also occur after abdominal surgery, typically pancreato-duodenectomy associated with injury of the main hepatic artery or some aberrant hepatic arteries, split liver transplantation, or chemoembolization or ablation of liver tumors, or might result from the surinfection of preexisting hepatic lesions, such as hepatic cysts, tumors (primary or secondary), or hydatid cysts (<2%) (*7*,*8*). When none of these mechanisms are found, pyogenic liver abscesses are considered cryptogenic (no obvious cause); such abscesses account for ≈20% of cases in industrialized countries (*9*–*12*).

In most instances, pyogenic liver abscesses are polymicrobial, and *Escherichia coli* is typically the most common pathogen involved (*13*). Since the 1990s, pyogenic liver abscesses caused by specific hypervirulent strains of *Klebsiella pneumoniae* have emerged as a major epidemiologic problem in Southeast Asia and now represent >80% of pyogenic liver abscesses in Asia (*11*,*14*,*15*). In the past decade, cases of hypervirulent *K. pneumoniae *pyogenic liver abscesses have been reported worldwide, including in Europe and North America, in patients with no travel history to Asia (*16*–*25*).

Typically, hypervirulent *K. pneumoniae *are responsible for severe monomicrobial cryptogenic pyogenic liver abscesses (*11*) often associated with unusual septic metastatic localizations, such as endophthalmitis or meningitis, in immunocompetent hosts. Colonies of hypervirulent *K. pneumoniae *grown on agar plates have a hypermucoviscous phenotype, as shown by a positive result for a string test (*11*). These strains express genes encoding for virulence factors, such as the hypermucoviscous phenotype (*prmpA*), iron acquisition systems (*iutA*,* kfu*,* ybts*), and the capsular serotypes K1 or K2 (*magA* and *wzi*) (*26*,*27*).

After recent observations of hypervirulent *K. pneumoniae *pyogenic liver abscesses at Hôpital Beaujon (Clichy, France) and other medical centers in France (*22*), we hypothesized that hypervirulent *K. pneumoniae *cryptogenic pyogenic liver abscesses might also represent an emerging disease in France. Thus, we retrospectively analyzed the characteristics of *K. pneumonia*e pyogenic liver abscesses in a monocentric cohort of pyogenic liver abscesses during 2010−2015 and compared the characteristics of cryptogenic and noncryptogenic *K. pneumoniae* abscesses.

## Methods

### Study Population

We conducted a retrospective, monocentric, cohort study at Hôpital Beaujon, a 500-bed tertiary care university hospital on the outskirts of Paris. This hospital specializes in digestive and liver diseases and is 1 of 5 major regional centers specializing in liver diseases; the hospital serves a population of 12 million persons (≈15% of the population of France). We reviewed all records from 2010–2015 in which a primary diagnosis was liver abscess. After records were made anonymous, we reviewed medical charts to confirm a diagnosis of liver abscess, which was defined by the association of typical clinical features (fever, abdominal pain); biologic abnormalities (inflammatory syndrome, increased bilirubin level); and lesions corresponding to an abscess observed by imaging (computed tomography scan, ultrasonography, or magnetic resonance imaging). Only patients with a microbiologically proven pyogenic abscess were included.

All medical records were reviewed by the same group of physicians, including clinicians (B.R., M.L.G., and A.L.) and a radiologist (M.R.), and cases were divided into 4 groups according to the suspected origin of the infection: biliary, portal, postprocedural, or superinfection of underlying liver diseases. In the absence of any of these conditions, pyogenic liver abscesses were considered cryptogenic. We then obtained *K. pneumoniae* pyogenic liver abscesses from each group and analyzed their epidemiologic, clinical, radiologic, and microbiological characteristics and their outcomes.

### Identification and Characterization of *K. pneumoniae* Isolates

For all patients included in this study, microbiological identification was available by blood cultures or cultures of abscess drainage. At the time of disease, all strains had been identified by using the API System (bioMérieux, Marcy l’Étoile, France) or matrix-assisted laser desorption-ionization/time-of-flight mass spectrometry (Bruker, Wissembourg, France), and antibiograms had been created. For this study, all available *K. pneumoniae* isolates underwent other phenotypic and molecular tests.

We assessed hypermucoviscosity by using the string test. A positive result for this test was defined as formation of a viscous string >5 mm when bacterial colonies are stretched on an agar plate (*28*). We determined capsular serotypes K1 or K2 and 8 virulence genes (a plasmid-borne regulator of the extracellular polysaccharide synthesis gene [*prmpA*], 4 iron-capture systems genes [aerobactin *iutA*, yersiniabactin *ybtS* *kfu*, and entrobactin *entD*], a capsular fucose production gene [*wcaG*] [*29*], an allantoin metabolism gene [*allS*], and a type-3 fimbrial adhesion gene [*mrkD*]) by using a multiplex PCR, as described (*30*). We performed multilocus sequence typing by using the international *K. pneumoniae* multilocus sequence typing scheme (http://bigsdb.web.pasteur.fr/).

### Statistical Analysis

We compared characteristics of *K. pneumoniae* cryptogenic and noncryptogenic pyogenic liver abscesses by using Student *t*-test or Wilcoxon rank-sum test for continuous variables and χ^2^ or Fisher exact tests for categorical variables, when appropriate. A p value <0.05 was considered significant. We performed statistical analyses by using Stata version 13 (StataCorp LLC, College Station, TX, USA).

## Results

**Figure 1:**
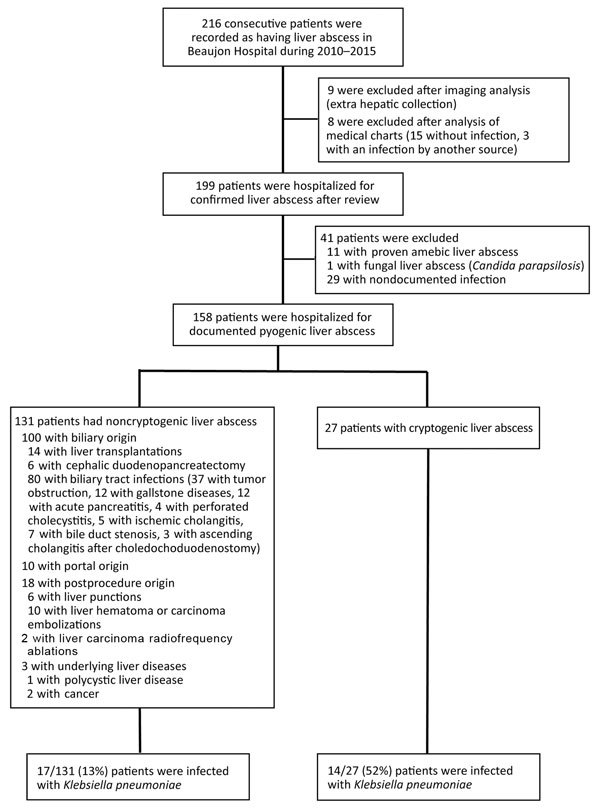
Flow chart for selection of 158 patients with microbiologically proven pyogenic liver abscesses and determination of *Klebsiella pneumoniae* infection, Hôpital Beaujon, Clichy, France, 2010−2015.

**Figure 2:**
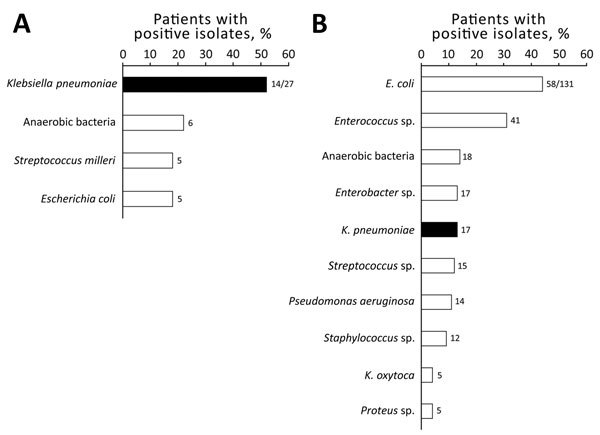
Bacteria isolated from patients with A) cryptogenic (n = 27) and B) noncryptogenic (n = 131) liver abscesses, Hôpital Beaujon, Clichy, France, 2010−2015. Black bars indicate *Klebsiella pneumoniae*. Values above bars indicate number of isolates. Differences for each bacterial species between cryptogenic and noncryptogenic abscesses were statistically significant for *K. pneumoniae* (p = 0.00005), *Enterococcus* species (p = 0.0001), *Staphylococcus *species (p = 0.0009), and *Enterobacter *species (p = 0.05). Five (18%) of 27 cryptogenic abscesses were polymicrobial, and 63 (48%) of 131 noncryptogenic abscesses were polymicrobial. *Enterococcus* species isolates were mainly *E. faecalis* (21 isolates) and *E. faecium* (21 isolates). For *Streptococcus* species isolates, 13/15 were *S. milleri*. *Staphylococcus aureus* represented 5 (42%) of 12 *Staphylococcus* species isolates.

A total of 216 patients had pyogenic liver abscesses during the study period. However, after analyzing all medical charts, we found that 199 pyogenic liver abscesses were definitively identified; these abscesses were further studied. Among the 199 abscesses, a microbiological diagnosis was available for 158 abscesses, which constitute the study cohort (Figure 1). Pyogenic liver abscesses were cryptogenic in 27 (17%) of 158 patients. *K. pneumoniae* was isolated from 31 (20%) of 158 patients and was more frequent in patients with cryptogenic pyogenic liver abscesses (14/27, 52%) than in those with noncryptogenic pyogenic liver abscesses (17/131, 13%; p = 0.00003). *K. pneumoniae* was the most common causative microorganism in the group with cryptogenic pyogenic liver abscesses (Figure 2).

We analyzed characteristics of patients having *K. pneumoniae* cryptogenic and noncryptogenic liver abscesses (Table 1). A total of 13/17 patients with *K. pneumoniae* noncryptogenic pyogenic liver abscesses had a biliary tract origin, and 2/17 showed development of pyogenic liver abscesses after liver transplantation (1 patient after cyst punction and 1 patient after chemoembolization). Healthcare-related infections (12/17 vs. 0/14; p<0.00001) and cancer (7/17 vs. 0/14; p<0.01) were significantly more frequent for patients with noncryptogenic pyogenic liver abscesses than for patients with cryptogenic pyogenic liver abscesses. We found a tendency toward higher C-reactive protein levels for patients with *K. pneumoniae* cryptogenic pyogenic liver abscesses. We observed 7 relapses (41%) for the 17 patients with *K. pneumoniae* noncryptogenic pyogenic liver abscesses, compared with none for the 14 patients with cryptogenic pyogenic liver abscesses (p<0.01) (Table 1).

**Table 1:** Characteristics of 31 patients with cryptogenic or noncryptogenic *Klebsiella pneumoniae* liver abscess, Hôpital Beaujon, Clichy, France, 2010−2015*

Characteristic	Cryptogenic liver abscess, n = 14	Noncryptogenic liver abscess, n = 17	p value
Median age, y	62	63	NS
Sex			
M	9 (65)	12 (71)	NS
F	5 (35)	5 (29)	NS
Ethnic group			
Caucasian	5 (36)	8(47)	NS
African	6 (43)	8(47)	NS
Asian	3 (21)	1 (6)	0.3
Healthcare related	0	14 (82)	<0.000005
Immunosuppression†	0	9 (53)	<0.002
Cancer	0	7 (41)	<0.01
Diabetes	7 (50)	7(41)	NS
Corticosteroids	0	2 (12)	NS
Concurrent condition		2 (14)	
Cirrhosis	0	2 (12)	NS
Renal insufficiency	2 (14)	1 (6)	NS
Heart failure	0	0	NS
Malnutrition	0	2 (12)	NS
Clinical features			
Fever	10 (71)	13 (76)	NS
Abdominal pain	6 (43)	8 (47)	NS
Severe sepsis	4 (29)	4 (24)	NS
Septic metastasis	3 (21)	0	0.08
Biological features, median (IQR)			
C-reactive protein, mg/L	229 (246)	96 (64)	NS
Bilirubin, mg/dL	14 (10)	22 (14)	NS
Morphologic features			
Multiple abscesses	4(29)	8 (47)	0.3
Right liver localization	12 (86)	10 (59)	0.1
Bacteriological features			
Polymicrobial	0	11 (65)	0.008
Positive blood culture	8/9	13/15	NS
Positive pus culture	11/11	11/12	NS
Antimicrobial drug-resistance phenotype			
Extended-spectrum β-lactamase	0/13	3/14	0.2
Drug susceptible	7/13	5/14	NS
Outcome			
Death	0	4(24)	0.08
Relapse	0	7(41)	<0.01
Follow-up, d, median (IQR)	177 (445)	391 (1,051)	NS

*Values are no. (%) or no. positive./no. tested unless otherwise indicated. NS, not significant (p>0.05). IQR, interquartile range (IQR is the difference between the 25th and 75th percentiles and is shown as a sample value).  †Some patients had several causes of immunosuppression.

In the cryptogenic group, 3 patients were of Asian origin, compared with 1 in the noncryptogenic group. Three patients had severe septic metastases. The first of these patients was a 64-year-old woman with diabetes from Sri Lanka who had K1-type *K. pneumoniae* pyogenic liver abscesses, endophthalmitis, and brain abscesses. She survived after enucleation treatment. The second patient was a 57-year-old man with diabetes from Morocco who had K2-type *K. pneumoniae* pyomyositis associated with pyogenic liver abscesses. He was cured after 6 weeks of treatment with antimicrobial drugs. The third patient was a 33-year-old man from France who did not have any previous medical condition, but in whom K1-type *K. pneumoniae* acute tibial periostitis associated with pyogenic liver abscesses developed. He was cured after 6 weeks of treatment with antimicrobial drugs.

### Microbiological Analysis

All 14 cryptogenic *K. pneumoniae* pyogenic liver abscesses but only 6/17 noncryptogenic cases were monomicrobial (p = 0.008). Among the 31 *K. pneumoniae* pyogenic liver abscesses, data for antimicrobial drug susceptibility were available for 13/14 cryptogenic isolates and 14/17 noncryptogenic isolates. A total of 23 (74%) of 31 isolates had been stored at −80°C and were further characterized: 13 (93%) of 14 from cryptogenic pyogenic liver abscesses and 10 (59%) of 17 from noncryptogenic pyogenic liver abscesses. We determined characteristics of these strains (Table 2).

**Table 2:** Characteristics of *Klebsiella pneumoniae* isolates from 13 cryptogenic and 10 noncryptogenic liver abscesses, Hôpital Beaujon, Clichy, France, 2010−2015*

Type of abscess and microbial type	K1/K2 serotype PCR result	Virulence genes								String test result	Sequence type
		*ybtS*	*mrkD*	*entD*	*prmpA*	*kfu*	*allS*	*iutA*	*wcaG*		
Cryptogenic											
Monomicrobial	K1	−	+	+	+	+	+	+	+	+	23
Monomicrobial	K1	+	+	+	+	+	+	+	+	+	23
Monomicrobial	K1	+	+	+	+	+	+	+	+	+	23
Monomicrobial	K1	+	+	+	+	+	+	+	+	+	23
Monomicrobial	K1	+	+	+	+	+	+	+	+	+	23
Monomicrobial	K1	+	+	+	+	+	+	+	+	+	23
Monomicrobial	K1	+	+	+	+	+	+	+	+	+	23
Monomicrobial	K1	+	−	+	+	+	+	+	+	+	23
Monomicrobial	K1	+	+	+	+	+	+	+	+	+	23
Monomicrobial	K1	+	+	+	+	+	+	+	+	+	23
Monomicrobial	K2	+	+	+	+	+	−	+	−	+	679
Monomicrobial	K2	+	+	+	+	−	–	+	−	+	86
Monomicrobial	K2	+	+	+	+	+	−	+	−	+	380
Noncryptogenic											
Monomicrobial	K2	+	+	+	+	−	−	+	−	+	65
Monomicrobial	–	−	+	+	−	−	−	−	−	−	495
Monomicrobial	–	−	+	+	−	−	−	−	−	+	2395†
Polymicrobial	–	+	+	+	−	−	−	−	−	−	17
Polymicrobial	–	−	+	+	−	−	−	−	−	−	323
Polymicrobial	–	+	+	+	−	−	−	−	−	−	45
Polymicrobial	–	−	+	+	−	+	−	−	−	−	15
Polymicrobial	–	−	+	+	−	+	−	−	−	−	188
Polymicrobial	–	−	+	+	−	−	−	−	−	+	788
Polymicrobial	–	+	+	+	−	+	−	−	−	−	405

*−, negative; +, positive. †New sequence type with housekeeping gene alleles *gapA*133, *inf*27, *mdh*19, *pgi*1, *phoE*47, *rpoB*135, and *ton*10.

All 13 *K. pneumoniae* isolates from cryptogenic pyogenic liver abscesses were confirmed to be hypervirulent *K. pneumoniae*; 10 had a positive string test result and a K1 (n = 10) or K2 (n = 3) capsular serotype. However, only 3/10 isolates from noncryptogenic pyogenic liver abscesses had a positive string test result (p<0.0005), and only 1 had a K2 serotype (p<0.00005). This K2 isolate had 3 virulence genes (*prmpa*, *iutA*, and *ybts*); 2 pyogenic liver abscesses in the drainage area of ​​an obstructed bile duct caused by pancreatic cancer developed in the patient infected with this strain.

We found the *prmpA* gene in all isolates from cryptogenic pyogenic liver abscesses but only in 1 isolate from noncryptogenic pyogenic liver abscesses (p<0.00005). Other virulence genes, such as *iutA*, *ybtS*, *kfu*, *wcaG*, and *allS*, were more common in isolates from cryptogenic pyogenic liver abscesses, and *entD* and *mrkD* were similarly present in both groups (Table 2). All K1 *K. pneumoniae* had sequence type (ST) 23. We further noted new alleles of the *gapA* and *rpoB* genes, which resulted in a new ST (ST2395) (Table 2).

*K. pneumoniae* isolates from cryptogenic pyogenic liver abscesses were less likely to be antimicrobial drug resistant than were *K. pneumoniae *isolates from noncryptgenic pyogenic liver abscesses. A total of 54% of the isolates were wild type, and no extended-spectrum β-lactamase (ESBL) was detected in the 13 strains from cryptogenic pyogenic liver abscesses compared with 36% wild-type strains and 21% ESBL-producing strains in the noncryptogenic group.

## Discussion

We studied 199 patients with liver abscesses managed in a single hospital during 2010−2015 and analyzed in detail 158 patients with microbiologically proven pyogenic liver abscesses. Although our study was retrospective, it had the advantage of particularly homogeneous management in a monocentric university hospital specializing in gastroenterology, liver diseases, and abdominal surgery. The main finding was that hypervirulent *K. pneumoniae* are now responsible for most cryptogenic liver abscesses in patients in the Paris region in France.

Pyogenic liver abscesses caused by hypervirulent *K. pneumoniae *represented 14 (8.9%) of 158 liver abscesses, which is more frequent than previously described in Europe (*16*). Although hypervirulent *K. pneumoniae *have only rarely been described in Europe and North America (*18*,*21*,*22*,*28*,*31*), our results indicate that hypervirulent *K. pneumoniae *pyogenic liver abscesses might be considered as an emerging infection in the Paris area and should always be considered in the diagnosis of patients with pyogenic liver abscesses of no obvious origin. This factor is a key point because infections are often severe, metastatic locations might impair prognosis (especially for meningeal or ocular involvement), and immediate treatment with antimicrobial drugs is required.

We found a relatively low proportion (17%) of cryptogenic pyogenic liver abscesses, which might be related to the conditions of our hospital, which is a highly specialized tertiary care hospital. However, similar rates of cryptogenic abscesses were reported in the United Kingdom (7/42, 16.7%) (*32*). This finding suggests that epidemic diffusion of hypervirulent *K. pneumoniae *in Europe has not yet reached its peak, in contrast to what was observed in other regions, because cryptogenic pyogenic liver abscesses now represent as many as 34% of pyogenic liver abscess cases in Australia (*33*) and 65% of pyogenic liver abscess cases in Asia (*34*).

All *K. pneumoniae* isolates from these cryptogenic abscesses were hypermucoviscous (defined as a positive string test result) and belonged to K1 or K2 serotypes, thus having the characteristics of hypervirulent *K. pneumoniae*. Similar to Ye et al. (*35*), we observed a clonal diffusion of ST23 K1-type hypervirulent *K. pneumoniae*, in contrast to K2-type hypervirulent *K. pneumoniae*, which belong to different sequence types (ST65, ST86, ST380, and ST679) (*35*).

One patient with noncryptogenic pyogenic liver abscesses was also infected with a strain harboring phenotypic and genotypic characteristics of hypervirulent *K. pneumoniae*. This finding indicated that hypervirulent *K. pneumoniae* might also be involved in noncryptogenic pyogenic liver abscesses.

From a clinical point of view, our description of hypervirulent *K. pneumoniae*−related pyogenic liver abscesses is consistent with previous reports. These abscesses were mostly monomicrobial, rarely harbored organisms that showed antimicrobial drug resistance, occurred in persons with few concurrent conditions, were not healthcare-related, and were more likely responsible for aggressive inflammatory disease with metastatic infectious locations (*17*,*36*,*37*). Unlike a previous report (*17*), diabetes did not appear to be a risk factor for hypervirulent *K. pneumoniae *pyogenic liver abscesses, and Asian patients did not represent the main ethnic group (only 21% in our study vs. 50% in cohorts in the United States). In addition, hypervirulent *K. pneumoniae*−related pyogenic liver abscesses were usually single and located in the right hepatic lobe (*17*).

Although hypervirulent strains were involved and metastatic locations were frequent, outcomes were more favorable than for patients with noncryptogenic *K. pneumoniae* pyogenic liver abscesses, probably because patients with hypervirulent *K. pneumoniae*−related pyogenic liver abscesses were less likely to be immunosuppressed or have cancer. Thus, host factors seem to outweigh bacterial determinants in the prognosis of pyogenic liver abscesses (*38*). However, a substantial mortality rate (4%–10%) caused by these hypervirulent strains occurred and should not be underestimated (*17*,*36*–*38*). As found in our study, antimicrobial drug resistance was rarely an issue for these hypervirulent strains. Nevertheless, ESBL- and carbapenem-resistant hypervirulent *K. pneumoniae *have been reported in Taiwan, China, India, and France and might represent an emerging problem in the future (*39*–*42*).

The microbiological definition of hypervirulent *K. pneumoniae *is still ambiguous because none of the phenotypic (string test) or genotypic (capsular serotype and virulence genes) tests alone is specific for hypervirulence (*43**–**46*). However, the combination of clinically aggressive monomicrobial cryptogenic pyogenic liver abscesses and isolation of positive string test−positive *K. pneumoniae* isolates with K1/K2 capsular serotypes and these genes involved in hypervirulence (e.g., *prmpA *and *iutA*) is highly suggestive of hypervirulent *K. pneumoniae*−related disease. We believe that it is the combination of this typical clinical and microbiological presentation that defines what might be called the hypervirulent *K. pneumoniae *syndrome.

Our study had several limitations. First, this study was monocentric and retrospective, which might limit its epidemiologic scope. Second, information concerning patients’ travel history was missing. Third, because our center is highly specialized in liver diseases, cryptogenic hypervirulent *K. pneumoniae *pyogenic liver abscesses might be underestimated, and cancer or postsurgical pyogenic liver abscesses are probably overrepresented.

As the main microorganism responsible for cryptogenic pyogenic liver abscesses, hypervirulent *K. pneumoniae *represents an emerging microorganism in the Paris area. The clinical and molecular differences between *K. pneumoniae* implicated in cryptogenic and noncryptogenic pyogenic liver abscesses confirm the correlation between clinical and microbiological virulence and define a hypervirulent *K. pneumoniae *syndrome. This finding is a major epidemiologic shift that should be considered in the diagnostic and therapeutic management of patients with pyogenic liver abscesses without underlying or obvious causes. Given the rapid increase in the prevalence of hypervirulent *K. pneumoniae *pyogenic liver abscesses in other parts of the world, it is possible that France and Europe might experience the same epidemic evolution as occurred in Asia in the early 2000s. Thus, clinicians need to be prepared for this possibility.

